# Long-Term Treatment of Thalidomide Ameliorates Amyloid-Like Pathology through Inhibition of β-Secretase in a Mouse Model of Alzheimer’s Disease

**DOI:** 10.1371/journal.pone.0055091

**Published:** 2013-02-06

**Authors:** Ping He, Xin Cheng, Matthias Staufenbiel, Rena Li, Yong Shen

**Affiliations:** 1 Center for Advanced Therapeutic Strategies of Brain Disorders, Roskamp Institute, Sarasota, Florida, United States of America; 2 Department of Neurology and Institute of Neurology, Huashan Hospital, Fudan University, Shanghai, China; 3 Novartis Pharm Ltd., Nervous System Research, Basel, Switzerland; 4 Center for Hormone Advanced Science and Education, Roskamp Institute, Sarasota, Florida, United States of America; Massachusetts General Hospital, United States of America

## Abstract

Thalidomide is a tumor necrosis factor alpha (TNFα) inhibitor which has been found to have abilities against tumor growth, angiogenesis and inflammation. Recently, it has been applied in clinic for the treatment of multiple myeloma as well as some inflammatory diseases. However, whether thalidomide has any therapeutic effects on neurodegenerative disorders, i.e. Alzheimer’s disease (AD) is not clear. AD is characterized by excessive amount of amyloid β peptides (Aβ), which results in a significant release of inflammatory factors, including TNFα in the brain. Studies have shown that inhibition of TNFα reduces amyloid-associated pathology, prevents neuron loss and improves cognition. Our recent report showed that genetic inhibition of TNFα/TNF receptor signal transduction down-regulates β amyloid cleavage enzyme 1 (BACE1) activity, reduces Aβ generation and improves learning and memory deficits. However, the mechanism of thalidomide involving in the mitigation of AD neuropathological features remains unclear. Here, we chronically administrated thalidomide on human APPswedish mutation transgenic (APP23) mice from 9 months old (an onset of Aβ deposits and early stage of AD-like changes) to 12 months old. We found that, in addition of dramatic decrease in the activation of both astrocytes and microglia, thalidomide significantly reduces Aβ load and plaque formation. Furthermore, we found a significant decrease in BACE1 level and activity with long-term thalidomide application. Interestingly, these findings cannot be observed in the brains of 12-month-old APP23 mice with short-term treatment of thalidomide (3 days). These results suggest that chronic thalidomide administration is an alternative approach for AD prevention and therapeutics.

## Introduction

Originally, thalidomide was introduced as an effective tranquilizer and painkiller that is associated with significant teratogenic property in human beings. It has been reported that thalidomide reduces the rate of TNFα synthesis through enhancing the degradation of transcript [1], [2]. As a well-known TNFα inhibitor, thalidomide has clinically been re-introduced in recent years [3]. Nowadays, the drug is used to treat the patients with erythema nodosum leprosum [4], [5] due to the inhibition property of inflammation and the subjects with multiple myeloma [6], [7], [8] because of anti-angiogenic activity by inhibiting cell proliferation of endothelial cells.

Inflammation in the brains has emerged as a significant contributor to the neurodegenerative process in AD [9]. TNFα is one of the most prominent pro-inflammatory cytokines and plays a central role in initiating and sustaining the cytokine cascade during inflammatory responses. TNFα is synthesized as a transmembrane 26-kDa precursor protein (pro-TNFα) which is proteolytically cleaved to a soluble 17-kDa TNFα. Subsequently, solube TNFα forms a non-covalently linked homotrimer. Both soluble and transmembrane-bound TNFα have biological functions by binding to two different receptor subtypes of TNF receptor I and II (TNFRI and TNFRII), respectively. In the brains, TNFα is primarily generated by microglia [10] and astrocytes [11]. In some circumstances some of neuron populations synthesize and secrete TNFα as well [12]. Elevated TNFα levels are observed in the serum [13], [14] and the post-mortem brains [15], [16] of AD patients as well as APP transgenic mice [17], [18], [19], [20]. The elevation is correlated with disease progression in patients with severe AD [14]. It has been reported that TNFα gene polymorphisms is associated with an increased risk of AD [21]. Microglia activation is associated with enhanced TNFα prior to symptomatic stages of AD pathology in transgenic AD mice [10]. Besides TNFα level increase, we also found that TNFRI levels are elevated in the brains of AD patients [22]. Hence, targeting TNFα/TNFRI signals may be a beneficial strategy in AD with neuroinflammation [23], [24].

Inhibiting TNFα ameliorates amyloid-associated pathology, prevents the progressive loss of neurons and at last improves cognitive deficits in AD [25], [26], [27]. Recently, we found that genetic deletion of TNFRI inhibits Aβ generation through decreasing BACE1 levels and activity [28], implicating TNFα/TNFRI/NF-κB signaling pathway in BACE1 regulation. Therefore, we wonder whether thalidomide could reduce amyloid loads by modulating BACE1. Here, we found that chronic administration of thalidomide could greatly decrease glial activation and Aβ generation in brains of APP23 transgenic mice. More interestingly, the decreased neuropathological effects by thalidomide are through inhibition of BACE1.

## Materials and Methods

### Animals

All animal experiments were performed in compliance with a protocol approved by the Institutional Animal Care and Use Committee (IACUC) of Roskamp Institute. APP23 transgenic (20 males and 20 females in each age group) and non-transgenic wild type (20 males and 20 females in each age group) genotypes in our experiment are on the C57BL/6 background, which were provided by Novartis Institute for Biomedical Research and the mice express mutated human βAPP (Swedish double mutation, KM670/671NL) under neuron-specific murine Thy-1 promoter element [29], [30]. APP23 and non-transgenic wild type mice were crossed and the progenies were genotyped and characterized as APP23 with PCR followed by Western blot for brain APP protein, resulting littermates used in experiments [28].

### Thalidomide Administration

APP23 transgenic mice used in this project express mutated human βAPP (Swedish double mutation) under neuron-specific murine Thy-1 promoter element. Aβ deposits or Aβ plaques start to appear in the APP23 mouse brain at 9 months old (an onset of visible plaque deposits) and there are tremendous amount of Aβ production/deposit and Aβ plaques in the APP23 mouse brains at 12 months old. Therefore, our strategy was to treat thalidomide from the beginning of AD-like pathology, which may be at a similar stage of “MCI” or “mild AD”. For observation of long-term effects on AD-like pathological formation, thalidomide was administrated from the age of 9th to 12th month (total three months). For the purpose of short-term observation, thalidomide was applied for 3 days at the age of 12 months old. Mice were intraperitoneally administered once a day either with a dose of 100 mg/kg thalidomide (Catalog: T144; Sigma-Aldrich) suspended in 0.5% w/v carboxymethylcellulose sodium (CMC, C9481, Sigma-Aldrich) in PBS or with 0.5% CMC alone [31], [32]. This dose of thalidomide was applied as a half of the quantity usually used in cancer-related studies in mice [33], which reduces potential side effects observed in long-term thalidomide treatment. The treatment protocol for thalidomide is well tolerated by the animals [32], [34]. At the end of the injection period, mice were perfused with PBS supplemented 10U heparin. The brains were withdrawn and the left half of the brains was fixed with 4% paraformaldehyde for histological analysis, and the right half was frozen on dry ice for biochemical analysis.

### ELISA

Aβ_1–40_ and Aβ_1–42_ ELISA quantification was performed as described previously [28], [35], [36]. The neocortex of experimental subjects was isolated and homogenized in M-PER mammalian protein extraction reagent (catalog: 78503, Thermo scientific) and centrifuged at 14,000 g at 4°C for 1 h. Protein concentration was measured by protein assays (Bio-Rad Laboratories) following manufacturer’s instruction. The pellet with insoluble Aβ was dissolved in 98% of formic acid and centrifuged at 4°C for 30 min. The supernatant from the pellet was collected for the assay of insoluble Aβ_1–40_ and Aβ_1–42_. The levels of Aβ_1–40_ and Aβ_1–42_ were measured with an Aβ_1–40_ and Aβ_1–42_ ELISA kit (KHB3481 and KHB3544, Invitrogen). The ELISA system has been extensively tested and no cross-reactivity between Aβ_1–40_ and Aβ_1–42_ was observed. The quantification of insoluble Aβ ELISA measurement was normalized to corresponding tissue protein concentration. Data were presented as Mean ± SD of four experiments.

### BACE1 Activity

An aliquot of brain homogenates was further lysed with a lysis buffer described as previously [28]. Briefly, BACE1 enzymatic activity was analyzed by using synthetic peptide substrates containing BACE1 cleavage site (BVI Substrate, a Lucifer Yellow labeled peptide, Catalog: #565781, Calbiochem). BACE1 substrate was dissolved in DMSO and mixed with HAc buffer (100 mM HAc and 100 mM NaCl, pH 4.5). An equal amount of protein was mixed with 100 µl of substrate. The fluorescence intensity was measured with a microplate reader (Bio-Rad laboratories) at an excitation wavelength of 430 nm and an emission wavelength of 520 nm. The average velocities were calculated and relative velocities were plotted in comparison with vehicle samples (100%).

### Western Blot

Western blot was performed as described previously [28]. The neocortex from mice (n = 10 each group, 5 males and 5 females) was homogenized in M-PER mammalian protein extraction reagent (catalog: 78503, Thermo Scientific) supplemented with Halt protease and phosphatase inhibitor single-use cocktail (Catalog: 78442, Thermo Scientific). The supernatants were directly separated on 8% SDS-PAGE and transferred to polyvinylidene fluoride (PVDF) membrane using wet transfer equipment at 90 mA overnight (Bio-Rad Laboratories). Following the transfer onto PVDF membranes a blockade with 5% dry milk was performed in Tris Buffer Saline (TBS). The membranes were incubated with primary antibodies overnight: rabbit polyclonal antibody against C-terminal fragment of APP (catalog: #171610, clone: 751–770, Calbiochem), monoclonal mouse anti-BACE1 antibody (MAB931, clone: 137612, 1∶1000, R&D Systems), rabbit anti-N-terminal BACE1 (B0806, clone: 485–501, Sigma-Aldrich), mouse anti-human soluble β APP (Swedish mutation) (sAPPβ, catalog: 10321, Clone: A61, 1∶1000, IBL-America), rabbit anti-insulin degrading enzyme (IDE, N-terminal 97–273, catalog: PC730, 1∶2000, Oncogene Research Products), rabbit anti-neprilysin (NEP, Neutral endopeptidase, MAB5458, 1∶2000, Millipore Bioscience Research Reagents), rabbit anti-presenilin 1 (PS-1, 1∶2000, gift by Dr. Selkoe), rabbit anti-APH-1 (1∶2000, gift by Dr. Yueming Li) and rabbit anti-nicastrin (**N1660, 1∶2000, Sigma-Aldrich**). Corresponding goat anti-mouse or rabbit IgG HRP-conjugated secondary antibodies (SC-2004 and SC-2055; Santa-Cruz Biotechnology, Santa Cruz, CA) were applied. The membranes were developed with SuperSignal West Femto Maximum Sensitivity Substrate (Catalog: 34095, Thermo Scientific) and the chemiluminescent image signal was detected and captured by ChemiDoc XRS (Bio-Rad Laboratories). After stripping in the strip solution, membranes were re-probed with a mouse anti-β-actin antibody (A1978; clone AC-15, Sigma-Aldrich). For quantification purposes, the densitometry of the protein signals was measured using Quantity One software (Version 4.6.0, Bio-Rad Laboratories). The ratio of protein signals versus (vs) corresponding β-actin signal was calculated and the results were expressed as density folds of the experimental group ratio to that of vehicle group, accordingly.

### Histology and Immunostaining Assay

The subject mice (n = 6 each group) were perfused via 0.1 M Phosphate Buffer (PB) supplemented with 10 U of heparin. The half brain was harvested and post-fixed in 4% paraformaldehyde (PFA). Serial sagittal sections (30 µm) were generated using Leica CM3000 cryostat. To observe the fibrillary aggregation of β-sheet amyloid, the sections were incubated in thioflavine S (T1892, 1∶5000, Sigma-Aldrich). To test Aβ accumulation as well as glial activation, immunostaining was performed as described previously [28], [37]. Sections were penetrated with 0.015% Triton X-100 and were blocked with 10% horse or goat serum. The primary antibodies were applied with monoclonal antibody against Aβ amino acid sequence 1–17 (MAB1560, clone 6E10, 1∶2000, Millipore Bioscience Research Reagents, Billerica, MA), rabbit anti-glial fibrillary acidic protein (GFAP) for test of astrocyte activation (Z0334,1∶5000, DAKO) and rat anti-CD45 for microglial activation (MCA1388, 1∶500, AbD, Serotec). Biotinylated secondary antibodies against rabbit IgG or mouse IgG were used (1∶1,000; Vecter Lab). Counter staining was performed with Mayer’s Hematoxylin Solution (MHS32, Sigma-Aldrich) for 1 min. Congo red binds to fibril proteins enriched in β-sheet conformation as a histological dye for amyloid detection [38]. To examine whether glial activation is associated with Aβ aggregation, Cong Red (0.5% w/v, Catalog: C6277, Sigma-Aldrich) was applied for 5 min.

### Quantification of Immunoreactive Structures

Quantification was carried out by an experimenter blind to the study as described previously [28]. Immunostaining was performed with sections per interval of 400 µm. A microscope (DMLS; Leica) with a 10× N PLAN and 20× and 40× PL FLUOTAR was used. Digitized images were captured with a DEI-470 digital camera (Optronics, Goleta, CA) on a Leica microscope (Leica, Germany). MagnaFire software (version 2.1C; Optronics) was used. The immunopositive structures of each section were counted with same parameter. In general, 9–11 sections through the hippocampus formation per mouse were calculated (n = 10 mice each group). The number of immune-positive structures was totalized and expressed per section.

### Statistical Analyses

Results were expressed as Mean ± SD. All analyses were performed using a software program (SPSS version 11.5.1; SPSS). Two groups were assessed using Student’s *t* tests. Three groups or more ware analyzed with variance models (ANOVA). The level of significance was *p*≤0.05.

## Results

### Thalidomide Decreases Glial Activation

**Figure 1:**
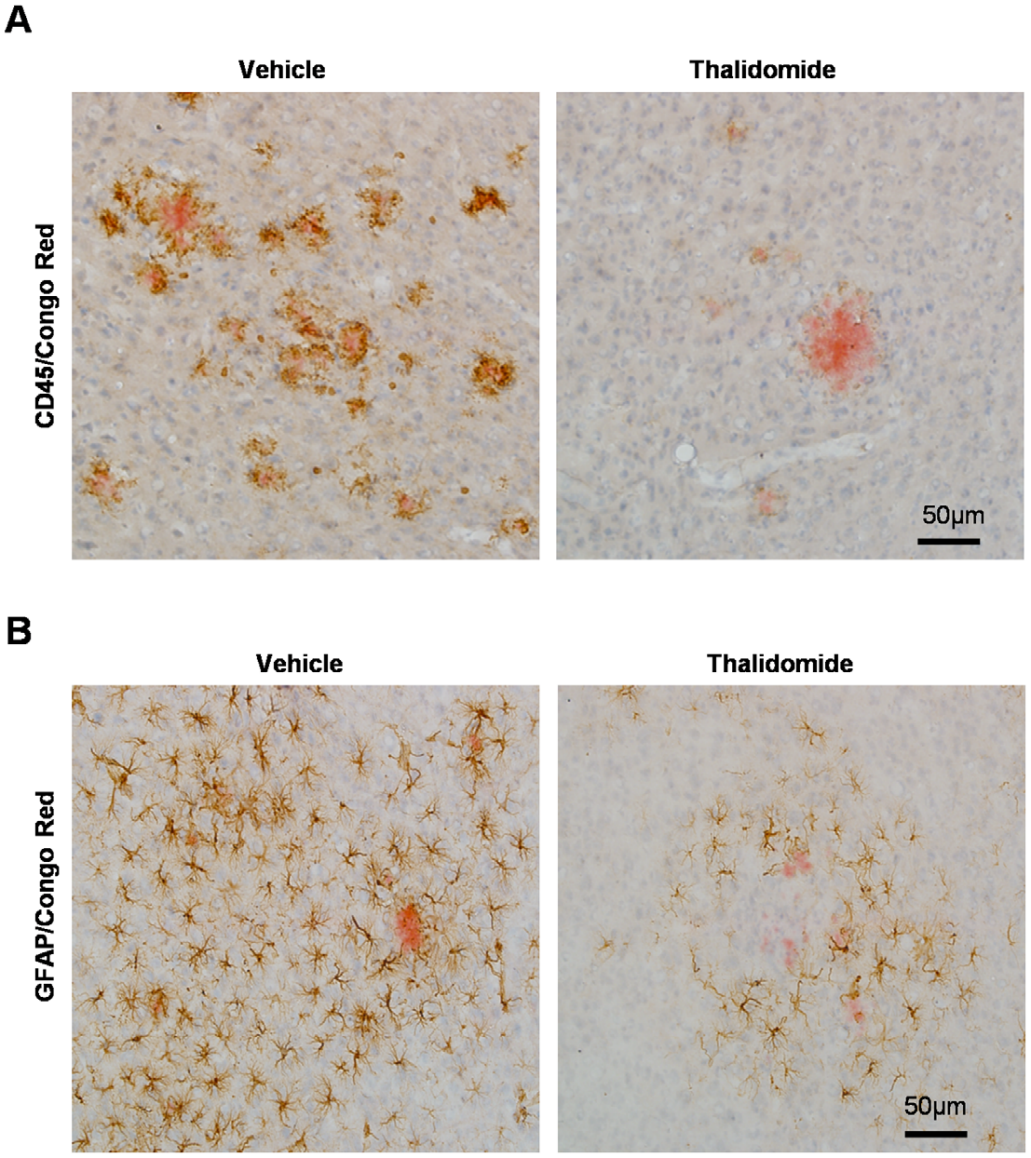
Thalidomide reduces glial activation. Senile plaques were demonstrated by Congo Red (Red). Representative images showed activated CD45-positive microglia around plaques. The number of activated microglia around similar size of plaques was obviously decreased with thalidomide administration compared to age-matched vehicle groups (**A**). Similarly, microphotographic images showed that thalidomide administration obviously decreased the number of GFAP-immunoreactive positive astrocytes around amyloid plaques (**B**). Counter staining was performed by haemotaxylin. Bars: 50 µm (**A, B**).

A large number of activated microglia and astrocytes around neuritic plaques is also a hallmark of Aβ neuropathological progression [39], [40], [41]. CD45 is a marker for microglia activation in response to the content of inflammation in the brains [39], [40], [41]. To observe microglia activation along with thalidomide administration in APP23 mice, immunostaining against CD45 was performed. Results showed a weak immunoreactivity and decreased microglial number around similar size of plaques, confirmed by Congo Red**,** in the neocortex in the presence of thalidomide compared to vehicles (**Fig. 1A** **)**. GFAP is well-characterized marker for astrocyte activation in the brains. To examine whether the thalidomide application could alleviate the astrocyte activation of APP23 mice, the immunostaining of GFAP was performed. We found activated astrocytes around plaques identified by Congo Red (**Fig. 1B**). The number of activated astrocytes around similar size of plaques was reduced in the brains of APP23 mice with thalidomide administration, in comparison with vehicle groups (**Fig. 1B**). These results indicate that chronic thalidomide administration could alleviate inflammation reaction in APP23 mice.

### Thalidomide Decreases β Amyloid Pathology

**Figure 2:**
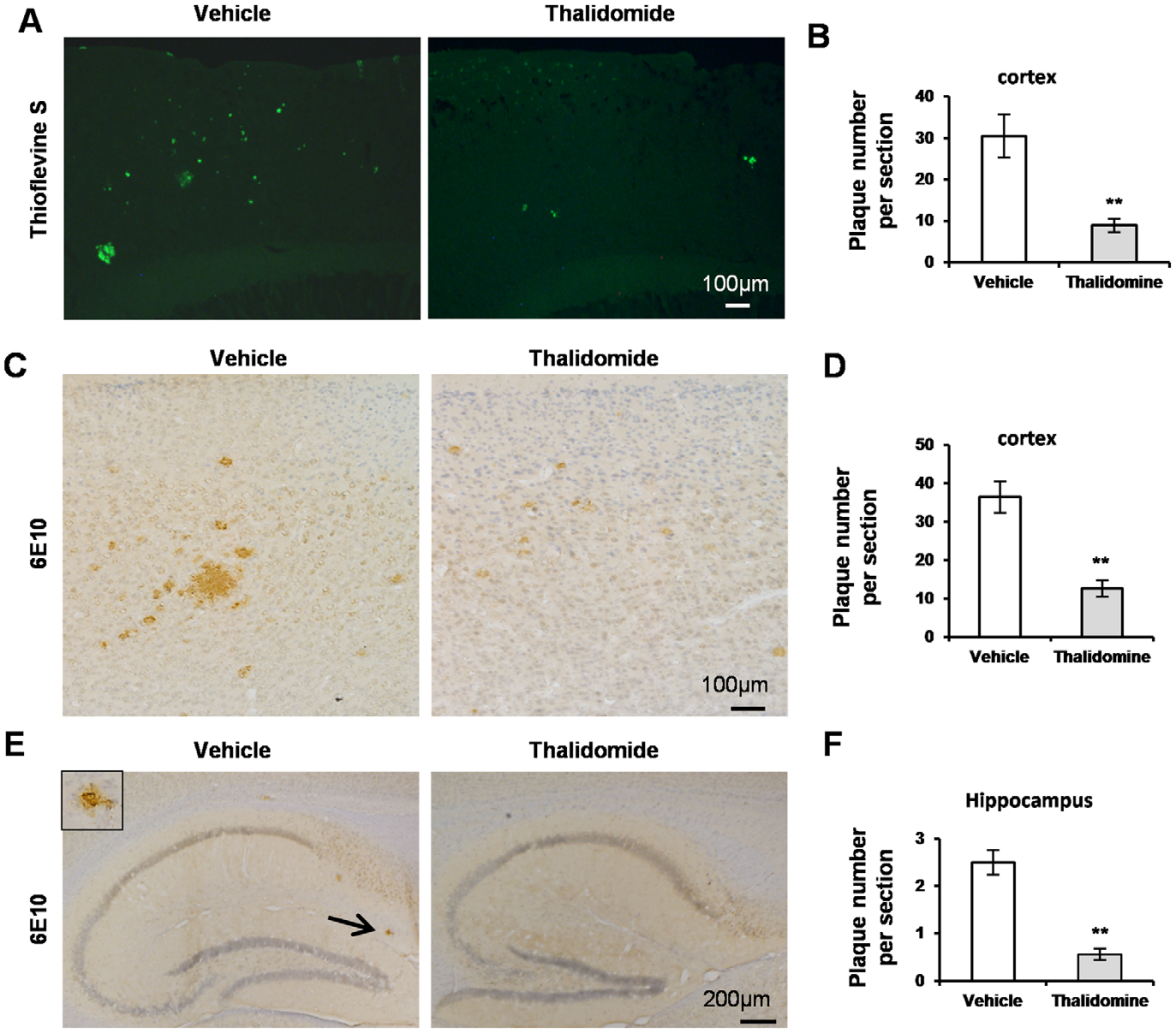
Thalidomide meliorates Aβ pathology. Representative images showed positive structures of thioflavine S staining, which confirms insoluble Aβ deposits, in the neocortex of 12-month-old APP23 mice with or without thalidomide application for 3 months (**A**). Thioflavine-positive plaques were counted and statistical analysis showed a significant decrease in the thioflavine-positive number of the neocortex along with thalidomide application vs vehicle group (Mean ± SD, **p<0.01, Student *t*-test, n = 10 each group) (**B**). Microphotographic images presented senile plaques which were confirmed by immunostaining of antibody against Aβ1-17 (Clone: 6E10) in the neocortex (**C**) and hippocampus (**E**). Counter staining was performed by haemotaxylin. Insert in (**E**) showed an amplified 6E10-positive plaque pointed by the arrow. Statistical analysis demonstrated a significant decrease in the number of 6E10-positive plaques in the neocortex (**D**) and hippocampus (**F**) (Mean ± SD, **p<0.01, Student *t*-test, n = 10 each group) following thalidomide administration. Bar: 100 µm (**A, C**), 200 µm (**E**).

To evaluate β amyloid (Aβ) pathology in the brains, thioflavine S, which binds to β sheet-rich fibril amyloid protein aggregates [42], was applied to observe whether a reduced protein aggregation could be seen in the brains in the presence of thalidomide. Results showed much less thioflavine S staining in thalidomide treated APP23 mice compared to vehicle groups (**Fig. 2A**). Accurate quantification in the cortex indicated 63% less number of plaques with thalidomide administration (**Fig. 2B**). To further confirm the results from thioflavine S staining, immunostaining was performed with antibody 6E10 recognizing Aβ1-17 fragment [43], [44]. Immune-positive plaques in the neocortex were shown in **Fig. 2C**. The plaque number was counted and a significant decrease was observed following thalidomide administration (**Fig. 2D**, **p<0.01). Similarly, representative images of the immunostaining against Aβ in the hippocampus were shown in **Fig. 2E**. In the group of thalidomide application the plaque number was reduced by 43% (**Fig. 2F**). These data strongly indicate that amyloid protein aggregation is alleviated in the presence of thalidomide.

### Thalidomide Decreases Aβ Levels

**Figure 3:**
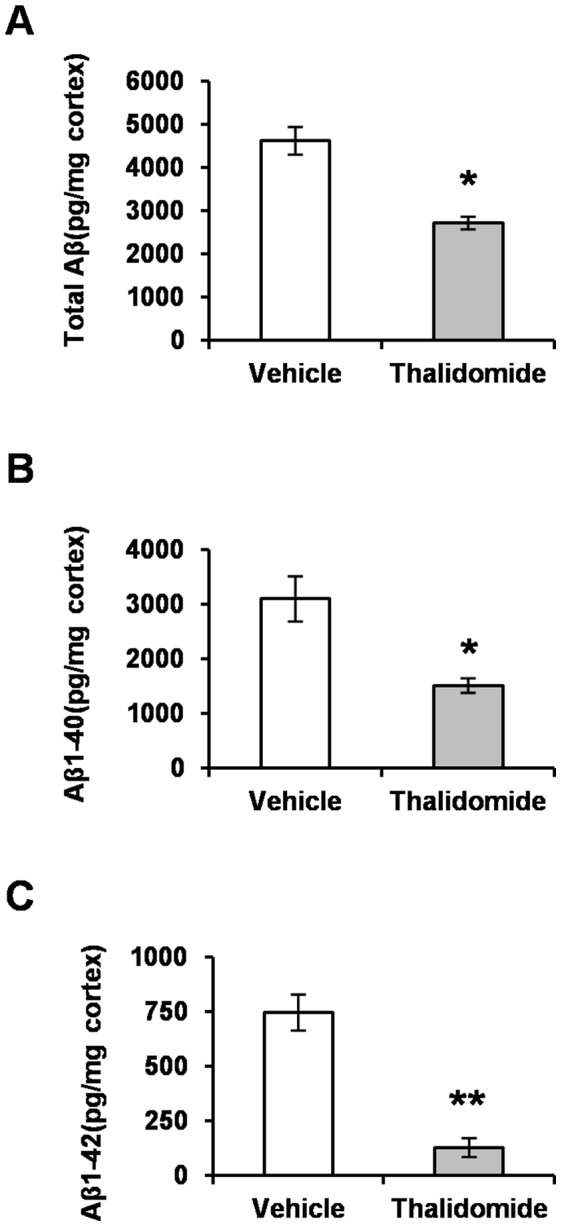
Thalidomide reduces Aβ load. ELISA analysis revealed a decrease in the amount of total Aβ (**A**), insoluble Aβ_1–40_ (**B**) and Aβ_1–42_ (**C**), which was calculated as picogram per milligram of protein in the neocortex of APP23 mice exposed 100 mg/kg of thalidomide for 3 months (Mean ± SD, *p<0.05, **p<0.01, Students *t*-test, n = 10 each group).

To further confirm the decrease in Aβ burden along with thalidomide administration, we wonder whether thalidomide reduces Aβ pathology by affecting Aβ generation. We measured total Aβ and Aβ_1–42_, Aβ_1–40_ levels, the two primary Aβ species in amyloid plaques [45], [46], [47] by sandwich ELISAs (n = 10 each group) [28], [36]. The pellets (detergent insoluble fraction) from brain tissue homogenization were re-suspended with formic acid. Quantitatively, ELISA results showed that total Aβ were significantly decreased by 41% (2718±145 pg/mg in the presence of thalidomide vs 4619±319 pg/mg in the vehicle groups) (**Fig. 3A**). Both insoluble Aβ_1–40_ and Aβ_1–42_ were significantly decreased by 51% (1513±133 pg/mg of thalidomide groups vs 3098±412 pg/mg of the vehicles, **Fig. 3B**) and by 83% (746±82 pg/mg vs 129±43 pg/mg, **Fig. 3C**), respectively. These results suggest that the amount reduction in Aβ_1–40_ and Aβ_1–42_ could account for the alleviated Aβ pathology in APP23 mice chronically treated with thalidomide.

### Thalidomide Down-regulates BACE1 and Lowers Amyloidogenic Processing of APP

**Figure 4:**
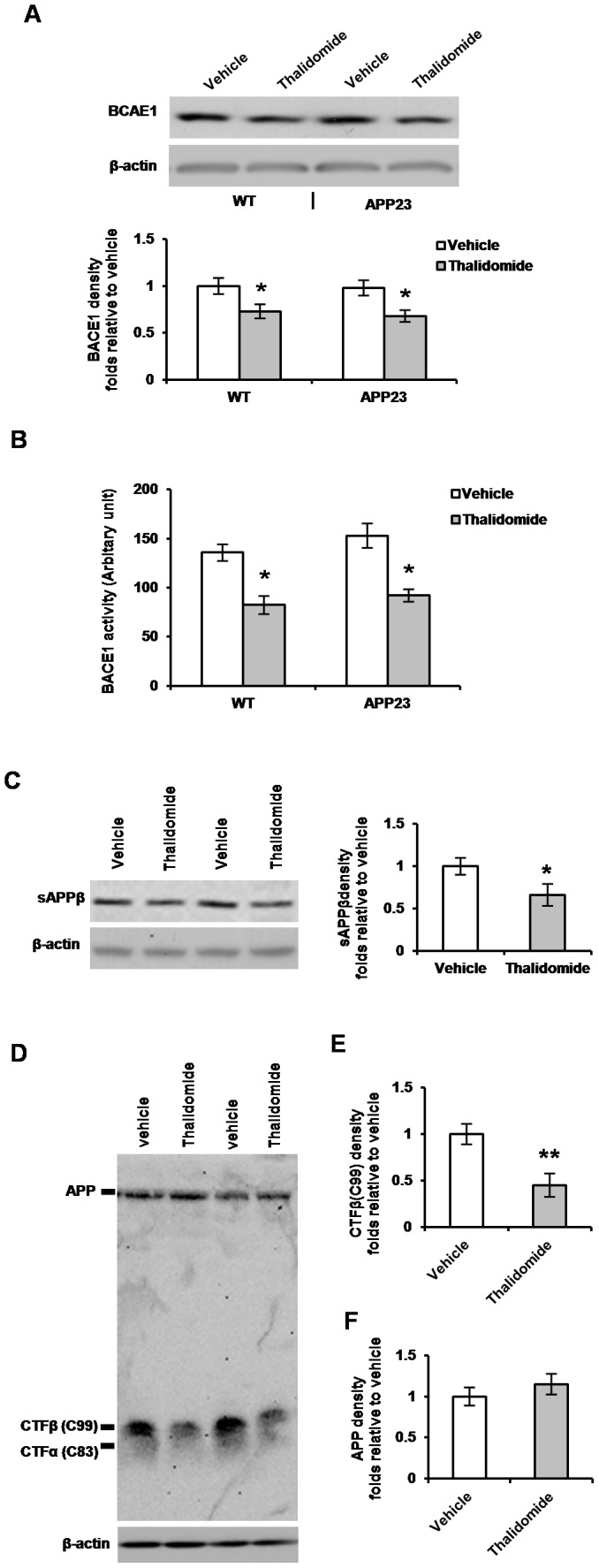
Thalidomide lowers BACE1 and reduces β-site cleavage of APP. Representative images of Western blots showed β-secretase enzyme BACE1 bands in WT and littermate APP23 mice with/without thalidomide administration (**A**) and a significant decrease in BACE1 amount was found with thalidomide application (**A**, Mean ± SD, *p<0.05, ANOVA test, n = 10 each group). BACE1 activity was normalized to the input protein amount and indicated as an arbitrary unit. The activity was measured and a lower activity of BACE1 was found with thalidomide application (**B**, Mean ± SD, *p<0.05, ANOVA test, n = 10 each group). Representative images of Western blots showed an amount decrease of sAPPβ secretion (**C**) and the density of bands significantly reduced in the thalidomide treated APP23 mice compared to littermate vehicle groups (**C,** Mean ± SD, *p<0.05, Students t-test, n = 10 each group). Microphotographic images of APP-CTF fragments showed an amount decrease of C99 following thalidomide administration (**D**). The density of bands was measured with a significant decrease in C99 fragments (**E**) but not significant changes of APP levels compared to vehicle groups (**F)** (Mean ± SD, **p<0.01, Students t-test, n = 10 each group).

β-Secretase (BACE1) is a type I transmembrane aspartyl protease, which is responsible for β-site amyloid-β precursor protein (APP) cleavage and is found to cleave APP at the N-terminal position of Aβ [43], [48], [49], [50], [51]. To examine whether the reduced amyloidosis in thalidomide-treated APP23 mice is caused by a reducing APP metabolism, Western blot was used to probe BACE1 expression level in the brains of age-matched WT and APP23 mice with/without thalidomide application (**Fig. 4A**). We found a significant decrease of BACE1 protein levels in the presence of thalidomide when compared to vehicle groups (**Fig. 4A**). Whether the BACE1 activity is also changed in APP23 mice in the presence of thalidomide still is unknown. We used an MCA-labeled BACE1 substrate [52], [53] to test the BACE1 activity (*n* = 10 for each group). We observed a significant decrease in BACE1 activity with thalidomide treatment (**Fig. 4B**). These results suggest that decreased BACE1 activity by thalidomide is due to a reduction in the protein levels.

The cleavage of APP occurs through BACE1 or α-secretase. Proteolytic enzyme BACE1 cleaves APP to produce a secreted soluble human mutant APPβ (sAPPβ) and carboxyl-terminal fragment β (CTFβ or C99) [47], [54]; α-secretase cleavage produces a secreted soluble APPα (sAPPα) and carboxyl-terminal fragment α (CTFα or C83). To examine whether the reduced amyloidosis in the presence of thalidomide may be caused by a reduction in APP metabolism, we at first observed the secretion levels of sAPPβ fragments by Western blot (**Fig. 4C**). We found a significant decrease of sAPPβ levels in the thalidomide-treated APP23 mice (**Fig. 4C**, *p<0.05). To further confirm that BACE1 cleavage was decreased following thalidomide administration, CTFβ (C99) fragment of BACE1 processing was tested by Western blot (**Fig. 4D**). The density of probing bands was measured. We found a significant reduction of C99 levels in APP23 mice treated with thalidomide compared to the vehicles (**Fig. 4E**). However, we did not find significant changes of APP protein levels between the groups of vehicle and thalidomide administration (**Fig. 4F**).

### Short-term Thalidomide has Little Effects on Glial Activation, Aβ Generation and BACE1 Activity

**Figure 5:**
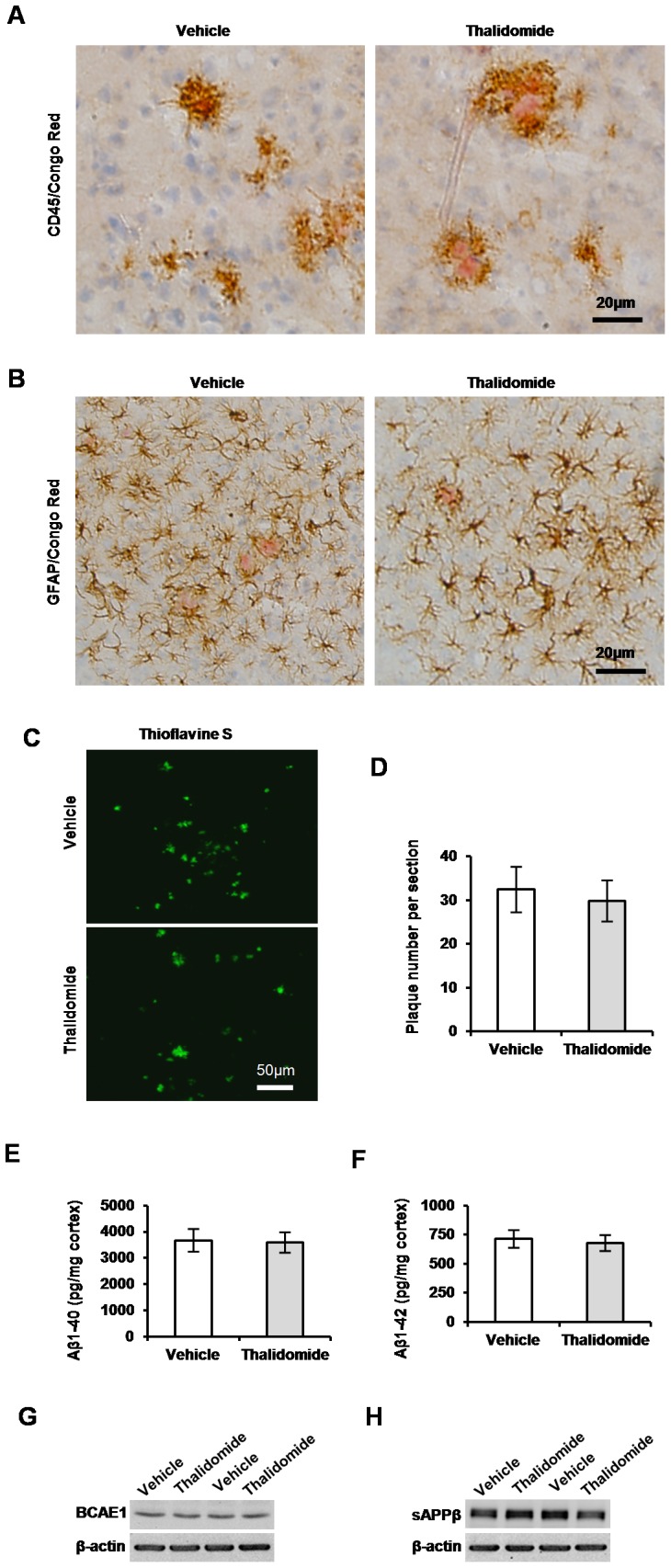
Short-term thalidomide has little effects on AD-like pathology and BACE1 regulation. Thalidomide was administrated for 3 days in 12-month-old APP23 mice. The examination was performed similar to that in the brains of APP23 mice treated with thalidomide for 3 months. Images showed that there is not obvious decrease in the number of activated CD45^+^ microglia (**A**) and GFAP^+^ astrocytes (**B**) around plaques in the neocortex (confirmed by Congo Red). Counter staining by haemotaxylin. Bars: 20 **Figure 7** **. Thalidomide does not affect amyloid degradation enzyme levels.** NEP and IDE, which are responsible for clearance of β amyloid, were measured by Western blot in WT and littermate APP23 mice in the absence or presence of thalidomide. Representative results were shown in (**A**) and (**C**), respectively. There were no significant changes in the amount of NEP (**B**) and IDE (**D**) expression between vehicle and thalidomide application (Mean ± SD, ANOVA test, *p*>0.05, n = 10 each group).

A reduced glial activation was observed with 3-month (long-term) administration of thalidomide (**Fig. 1**). Whether the activation decrease could occur with a short-term application of thalidomide is still not clear, we intraperitoneally administrated thalidomide with the same dose once a day for 3 days in 12-month-old APP23 mice. Similarly, the activation was observed with immunostaining of antibody against CD45 (microglia) and GFAP (astrocyte). Results showed no obvious different activation of either microglia (**Fig. 5A**) or astrocytes (**Fig. 5B**), suggesting that short-term administration of thalidomide could not help inflammatory reduction as we observed by long-term administration of thalidomide (**Fig. 2**). Moreover, we further determined whether a short-term application of the drug could decrease Aβ plaque number. Following 3-day administration, the evaluation of plaque number was performed with thioflavine S staining (**Fig. 5C**) and we did not find a significant difference of plaque number in the brains in the presence of acute thalidomide treatment vs vehicle groups (**Fig. 5D**). Although no significant decrease in plaque number, there is a possibility that a decreased amount of Aβ burden might still occur. Both Aβ_1–40_ and Aβ_1–42_ levels was measured by sandwich ELISAs (n = 10 each group) [28], [36]. Results showed no significant decrease in insoluble levels of both Aβ_1–40_ (**Fig. 5E**) and Aβ_1–42_ (**Fig. 5F** **)** with the short-term presence of thalidomide vs vehicle groups.

To test whether short-term thalidomide treatment can alter BACE1 expression, western blot was performed. Expectedly, we did not find any changes of BACE1 protein levels in the presence and absence of short-term thalidomide (**Fig. 5G**). To further confirm the result of not changing BACE1 activity with the short-term thalidomide application, we examined the secretion levels of sAPPβ fragments to observe the β-site cleavage of APP. Similarly, we did not find a significant decrease of sAPPβ levels in the short-term thalidomide-treated APP23 mice (**Fig. 5H**). These results suggest that thalidomide is not directly involved the modulation of BACE1.

### Thalidomide has Little Effect on γ-secretase Components

**Figure 6:**
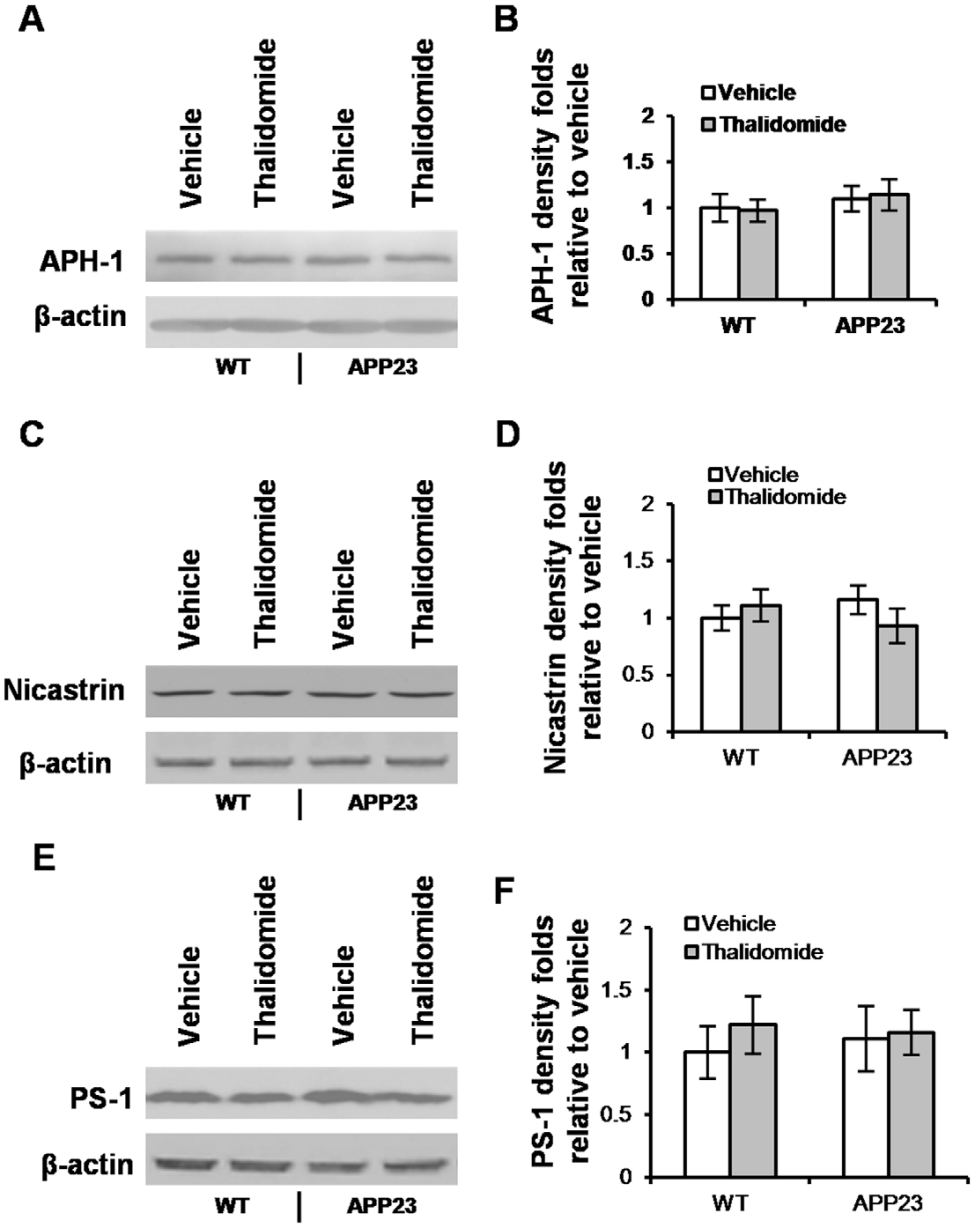
Thalidomide has little affect γ-secretase components. The γ-secretase components were probed with Western blot in WT and littermates APP23 mice with/without thalidomide administration. Representative images showed APH (**A**), Nicastrin (**C**) and PS-1 (**E**). Density analyses showed no significant changes of APH-1 levels (**B**), nicastrin (**D**) and PS-1 (**F**) with thalidomide application compared to vehicle groups (Mean ± SD, ANOVA test, *p*>0.05, n = 10 each group).

Carboxyl-terminal fragment (CTFβ or C99) of BACE1 processing can be further cleaved by γ-secretase, giving rise to Aβ [55]. Next, we examined the expressions of γ-secretase components: APH-1, nicastrin and PS-1, which cleave the C-terminals of Aβ. The protein expression was probed by Western blot (**Fig. 6A, C, E**) and we did not observe the obvious changes of APH-1, nicastrin and PS-1 expression in the presence of thalidomide (**Fig. 6B, D, F**) compared to responding vehicle groups, respectively.

### Thalidomide has Little Effect on Aβ Clearance Enzymes

**Figure 7:**
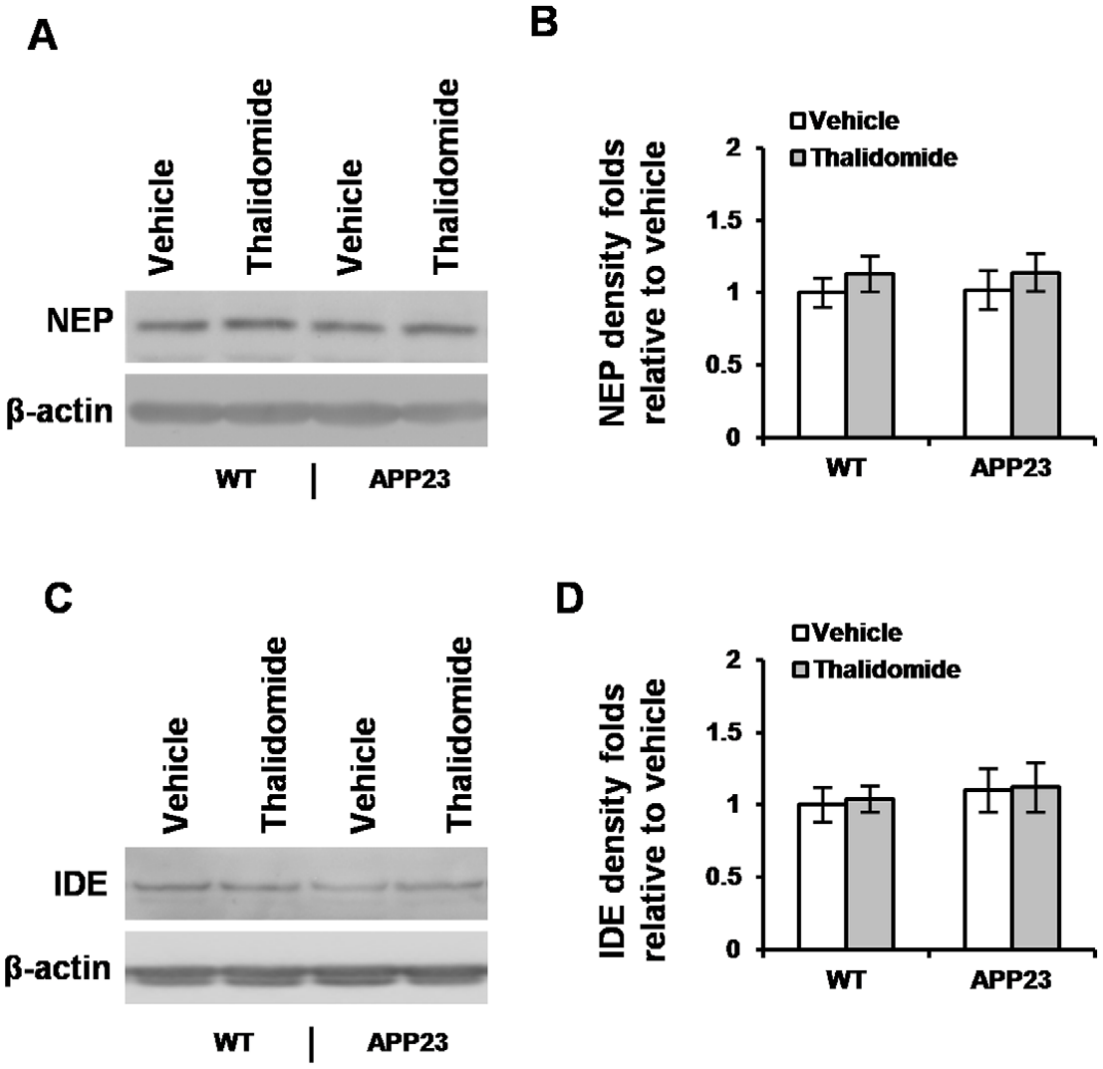
Thalidomide does not affect amyloid degradation enzyme levels. NEP and IDE, which are responsible for clearance of β amyloid, were measured by Western blot in WT and littermate APP23 mice in the absence or presence of thalidomide. Representative results were shown in (**A**) and (**C**), respectively. There were no significant changes in the amount of NEP (**B**) and IDE (**D**) expression between vehicle and thalidomide application (Mean ± SD, ANOVA test, *p*>0.05, n = 10 each group).

Thalidomide-induced Aβ reduction could also be due to an increase in Aβ degradation/clearance activity instead of Aβ production. The enzymes, insulin degradation enzyme (IDE) and nerilysin (NEP), which are relevant to Aβ degradation and clearance [56], were assessed. Western blot analyses did not show significant differences in either IDE or NEP levels between the presence of thalidomide and vehicle groups (n = 10 in each group) (**Fig. 7A–D**). The results indicate that thalidomide-induced reduction of Aβ levels is not associated with Aβ clearance and degradation of enzymes IDE and/or NEP.

## Discussion

In this study, this is the first report that long-term treatment of thalidomide could decrease activated cell number of microglia and astrocytes, which is consistent with previous report [57]. The activated inhibition of glial cells might be due to a decreased stimulation by downgrading Aβ deposits or by thalidomide-lowering TNFα levels. However, the reduced glial activation cannot be observed following a short-term treatment of thalidomide. It is postulated that with short-term treatment of thalidomide (1) there is no significant decrease in Aβ accumulation and therefore the stimulation by Aβ cannot be reduced; (2) the existence of glial activation induced by Aβ cannot be inactivated because of reduced TNFα levels caused by thalidomide.

Meanwhile, Aβ levels was reduced with chronic thalidomide treatment in this study, consistent with the recent report that 3,6′-dithiothalidomide, an isosteric analog of thalidomide, slows Aβ amount in neuronal cytoplasma of AD transgenic mice for 24 days [25], [26]. Senile neuritic plaques are a hallmark of AD brains [47], [58]. Here, we observed a decreased number of Aβ deposit plaques with chronic application of thalidomide but not with a short-term treatment. It indicates that thalidomide needs to be applied at a long term for preventative and therapeutic purposes.

BACE1 is a stress-response protein [59]. We [52], [53] and other groups [60], [61] found an increased BACE1 levels and/or activity in the brains of AD patients. BACE1 activity is also up-regulated by various factors, such as age, a primary risk factor for AD [62], inflammatory cytokine interferon γ [63], oxidative stress NO [64] and free radicals [65]. In the present study, we further demonstrated that inhibition of TNFα by thalidomide administration lowers BACE1 levels and activity and therefore ameliorates amyloid pathology. However, we did not find the down-regulation of BACE1 and its cleavage following a short-term treatment of thalidomide. It suggests that thalidomide regulates BACE1 through at least a modulator and plays the role by an indirect mechanism. Our previous experiments showed that *TNFRI* deletion could directly down-regulate BACE1 transcription through NF-κB [28], [52], [66]. We also found TNFRI level increase in the brains of AD patients [22]. These results strongly indicate that TNFα/TNFRI is involved in the up-regulation of BACE1 activity. Here we cannot exclude the possibility that the down-regulation of BACE1 activity induced by long-term thalidomide treatment results from a reduction in BACE1 protein levels. Further activity assay is needed to base on the equal levels of BACE1 protein instead of total proteins extracted from the brains of thalidomide treatment and vehicles.

BACE1-cleavage of APP is the rate-limiting step in Aβ production and pathogenesis of AD brains [46], [67]. Modulation in these BACE1-regulating proteins leads to changes in Aβ levels and pathogenesis in the brains of AD patients. Thus, BACE1 has been considered as a prime target for Aβ-lowering strategy in the prevention and intervention of AD. Besides searching for the inhibitors that directly target BACE1 [68], [69], targeting BACE1 modulators may be an alternative path to the therapeutics of AD.

Thalidomide is an immunomodulatory drug which is a brain-permeant small molecule inhibitor of TNFα [1], [2]. The inflammation inhibition of thalidomide extends survival in a transgenic mouse model of amyotrophic lateral sclerosis [70]. Regarding the little effects of thalidomide on γ-secretase as shown in the present study, *in vitro* studies demonstrated that the increase of γ-secretase activity requires up-regulation of four components: PS-1, APH-1, nicastrin and pen-2 in cell culture [71]. Our results *in vivo* showed that thalidomide has little effects on the expressions of three components: PS-1, APH-1, nicastrin in transgenic APP23 mice. The experiment did not include the observation of Pen-2 protein levels with thalidomide treatment. Even though Pen-2 could potentially be regulated by thalidomide, it would not change γ-scretase activity *in vivo*.

Meanwhile, our experimental results showed no significant differences in terms of the responses (BACE1 and Aβ) to thalidomide treatment in the brains between males and females of APP23 mice (data not shown). Moreover, it has been reported that thalidomide might partially prevent recognition impairment by Aβ toxicity [72]. In the present study we revealed that chronic administration of thalidomide dramatically decreased glial activation and Aβ neuropathology in the brains of an AD-like transgenic mouse model. The thalidomide-induced Aβ load reduction was caused by inhibition of BACE1. Re-introduction of thalidomide might ignite a promising aspect in immunological and inflammatory diseases such as neurodegenerative diseases [25], [73]. This is one of significances of using thalidomide as a potential treatment for AD. Our recent NIH supported phase II clinical trial by using thalidomide to treat AD patients is on-going [74]. If the clinical trial of thalidomide in AD patients works, it would provide an alternative approach to treat AD. Regarding the side effects of thalidomide, especially the issues for pregnant women, AD patients are selected at above 70 years old.
